# The Pure Milk Bills

**Published:** 1914-07-11

**Authors:** 


### THE PURE MILK BILLS.

At a meeting of the Parliamentary Committee on Food and Health, held at the House of Commons, and presided over by Mr. Charles Bathurst, M.P., the following resolution was moved by the Chairman, seconded by Sir W. Phipson Beale, M.P., and carried unanimously : "The Parliamentary Committee on Food and Health cordially supports the Milk and Dairies Bill and Milk and Dairies (Scotland) Bill as measures designed to secure to the community an unrestricted supply of pure milk, with the minimum amount of interference with the producer. While recognising them to be capable of improvement in some details, it earnestly deprecates the insistence on drastic amendments as calculated gravely to imperil the passage of Bills long overdue and of vital importance to the health of the people, and especially of the children. The Committee trusts, however, that a clause sanctioning the sale of 'certified milk,' which in the United States has played so conspicuous a part in raising the milk standard and informing public opinion in favour of clean milk, will, by general consent and in the interests alike of producers and consumers, be included in both measures."

